# The weighted cumulative exposure method and its application to pharmacoepidemiology: A narrative review

**DOI:** 10.1002/pds.5701

**Published:** 2023-09-25

**Authors:** Thu‐Lan Kelly, Amy Salter, Nicole L. Pratt

**Affiliations:** ^1^ Quality Use of Medicines and Pharmacy Research Centre, Clinical and Health Sciences University of South Australia Adelaide Australia; ^2^ School of Public Health The University of Adelaide Adelaide Australia

**Keywords:** cumulative exposure, pharmacoepidemiology, time‐varying exposure, weighted cumulative exposure

## Abstract

**Purpose:**

The weighted cumulative exposure (WCE) method has been used in a number of fields including pharmacoepidemiology where it can account for intensity, duration and timing of exposures on the risk of an outcome. The method uses a data driven approach with flexible cubic B‐splines to assign weights to past doses and select an aetiologically appropriate time window. Predictions of risk are possible for common exposure patterns encountered in real‐world studies. The purpose of this study was to describe applications of the WCE method to pharmacoepidemiology and assess the strengths and limitations of the method.

**Method:**

A literature search was undertaken to find studies applying the WCE method to the study of medicines. Articles published in PubMed using the search term ‘weighted cumulative exposure’ and articles citing Sylvestre et al. (2009) in Google Scholar or Scopus up to March 2023 were subsequently reviewed. Articles were selected based on title and review of abstracts.

**Results:**

Seventeen clinical applications using the data‐driven WCE method with flexible cubic splines were identified in the review. These included 3 case–control studies and 14 cohort studies, of which 12 were analysed with Cox proportional hazards models and 2 with logistic regression. Thirteen studies used time windows of 1 year or longer. Of 11 studies which compared conventional models with the WCE method, 10 (91%) studies found a better fit with WCE models while one had an equivalent fit. The freely available ‘WCE’ software package has facilitated the applications of the WCE method with flexible cubic splines.

**Conclusions:**

The WCE method allows additional insights into the effect of cumulative exposure on outcomes, including the timing and intensity (dose) of the exposure on the risk. The flexibility of the method is particularly well suited to studies with long‐term exposures that vary over time or where the current risk of an event is affected by how far the exposure is in the past, which is difficult to model with conventional definitions of exposure. Interpretation of the results can be more complex than for conventional models and would be facilitated by a standardised reporting framework.

**Key Points**

- The weighted cumulative exposure (WCE) method can model associations between exposures and outcomes which are dependent on dose, duration and recency of use but is more complex to interpret than conventional definitions of exposure.
- The method can be used to predict the risk of the outcome for clinically relevant patterns of exposure.
- The method is suitable for research questions which investigate exposures that may have different effects on the outcome depending on how far they are in the past.
- Data‐driven WCE models have been found to provide a better or similar fit to compared with conventional models.
- Statistical software implementing the method is freely available via the R package ‘WCE’. Currently only time to event outcomes using Cox proportional hazards models are available in the package.

## 1 INTRODUCTION

Investigations of risks for adverse effects of medicines using observational administrative databases can be challenging when there is complexity in time varying intensity and exposure patterns.^1^ Conventional or traditional models use either fixed in time summary measures for exposure such as dose at cohort entry or ‘ever use’, or simple time‐varying exposure metrics like ‘current use’ or mean dose in the recent past.^2^ Methods that incorporate a summary of duration, intensity and timing have been proposed as best practice to account for the real‐world effect of exposure.^3^, ^4^

Definitions of cumulative exposure based simply on summing past doses implicitly assume that all exposures in the past have the same effect on the current risk of an outcome, including after exposure has stopped. This may not reflect the pharmacological properties of medicines or aetiological risk of an outcome in many pharmacoepidemiology applications. To address this limitation, past exposures may be assigned weights which reflect the timing of an exposure on the current risk.

In decades prior to 2006, several authors proposed to account for the potential complexity in exposure‐outcome associations by modelling exposure as the cumulative effect of past doses weighted by recency^3^, ^5^, ^6^ or accounting for latent effects.^7^ These models have been used for occupational and environmental exposures, such as arsenic,^5^ asbestos^3^ or radon.^7^ Abrahamowicz et al.^8^ proposed the weighted cumulative exposure (WCE) method for particular use in pharmacoepidemiology with pre‐defined weight functions that assigned weights to past doses, where weights were estimated in pre‐defined risk periods based on drug half‐life, with doses further in the past assigned lower weights. Hauptmann et al.^4^ and Sylvestre and Abrahamowicz^9^ modelled cumulative exposure over aetiologically relevant time windows using flexible cubic splines to computationally estimate weights based on the data, rather than being defined a priori. This approach has been shown to provide a better fit relative to conventional models.^10^

Applications of WCE models using flexible cubic splines in pharmacoepidemiology have increased in recent years likely due to the development of an R package ‘WCE’ by Sylvestre et al.^11^ The software package, including the source code, is openly available for estimating the weight function with flexible cubic B‐splines to within a Cox proportional hazards model. Since the WCE models account for varying intensity, timing and duration of exposure, predictions for different clinically relevant patterns of medicine dose are possible. For example, Dixon et al.^2^ used the method to predict infection risk for common patterns of glucocorticoid use in patients with rheumatoid arthritis. Since the added complexity of the WCE method allows for a more comprehensive interpretation compared to conventional models, we conducted a narrative literature review in order to (1) describe the evolution of the WCE methodology, including strengths and limitations of the method, and its benefit compared to traditional models of exposure and (2) review applications of the WCE method in pharmacoepidemiology with safety or effectiveness outcomes.

## 2 METHODS

A literature review was conducted by searching journal articles indexed by PubMed with the term ‘weighted cumulative exposure.’ In addition, we identified studies that cited Sylvestre and Abrahamowicz,^9^ the developers of the R package ‘WCE’^11^ who requested that users of the package cite their publication if reporting results from the software. Protocols, preprints, conference abstracts, commentaries or opinion pieces and applications outside of pharmacoepidemiology, such as nutritional or environmental exposures, were excluded. Articles published up to March 1, 2023 were selected for review using the title and abstract. Articles were classified into two types depending on their primary purpose: (1) methodological development; or (2) applications to pharmacoepidemiology with safety or effectiveness outcomes. The application studies were summarised with information about the cohort (including country and sample size), analysis type (nested case–control design or survival analysis), exposure and outcome. Assessment was made of the strengths and limitations of the method as discussed in the literature.

## 3 RESULTS

**FIGURE 1:**
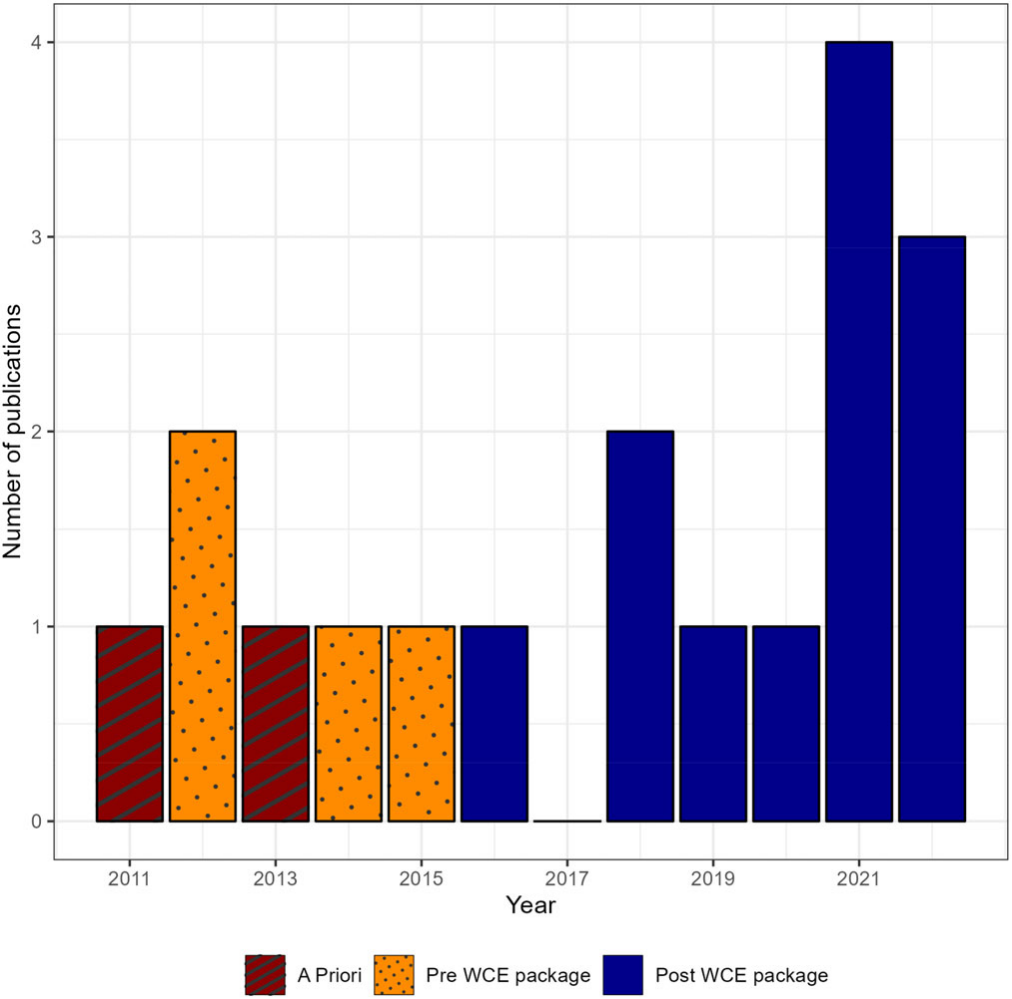
Number of clinical applications per year using the WCE method: weight function defined a priori (red) and with flexible cubic splines before (orange) and after (blue) availability of the ‘WCE’ R package.

From 42 studies identified through PubMed, 28 original articles met the inclusion criteria. There were three initial studies published between 2006 and 2012 that introduced and validated the WCE method and six further studies which primarily described methodological extensions to the method. There were 19 pharmacoepidemiologic applications, published between 2011 and 2022 (Table 1). Use of the method notably increased after the ‘WCE’ R package was released in 2015 (Figure 1).

**TABLE 1:** Applications of the WCE method to pharmacoepidemiology.

Authors	Study design/analysis	Cohort/country (*N*)	Outcome(s)	Exposure	Exposure time window	WCE better than conventional?
Weight function defined a priori (Abrahamowicz et al.^8^)						
Avina‐Zubieta et al.^35^	Cohort/survival with PS^a^	RA^b^/Canada (7051)	Cerebrovascular disease	Glucocorticoids		No
Avina‐Zubieta et al.^36^	Cohort/survival with PS^a^	RA^b^/Canada (8384)	Acute myocardial infarction	Glucocorticoids		No
Data driven weight function (Sylvestre and Abrahamowicz^9^)						
Sylvestre et al.^30^	Cohort/survival	Elderly/Canada (23 765)	Fall related injuries	Benzodiazepines	60–180 days	For some benzodiazepines
Dixon et al.^2^	NCC/CL^d^	RA^b^/Canada (11 682)	Infection	Glucocorticoids	3 years	Yes
Xiao et al.^18^	Cohort/survival with IPTW^e^	HIV^f^/Switzerland (11 625)	Cardiovascular disease	Didanosine	30 months	Yes
Moura et al.^28^	Cohort/survival	RA^b^/Canada (11 333)	Joint replacement	DMARDs	1 year	Not stated
Movahedi et al.^29^	Cohort/survival	RA^b^/UK (21 962) and USA (12 657)	Diabetes mellitus	Glucocorticoids	1 year	Yes
Bally et al.^19^	NCC/CL^d^	Elderly/Canada (233 816)	Acute myocardial Infarction	NSAIDs^g^	90 days	Equivalent
Louveau et al.^23^	Cohort/survival	RA^b^/France (403)	Radiographic progression of RA at 5 years follow‐up	Oral corticosteroids/DMARDs^c^	18–60 months	Yes
Weir et al.^31^	Cohort/survival	Type 2 diabetes/USA (7620)	Heart failure exacerbation	Metformin	30 days	Yes
Dankner et al.^24^	Cohort/survival	Diabetes/Israel (2 186 196)	Cancer	Metformin	10 years	Only WCE used
van Gaalen et al.^20^	NCC/CL^d^	Ciprofloxacin users/Netherlands (1139)	Ciprofloxacin resistance	Ciprofloxacin	90 days	Yes
Guilloteau et al.^27^	Cohort/survival	Metastatic colorectal cancer (PRODIGE9 RCT)/France (382)	All‐cause mortality	Bevacizumab	120 days	Yes (equivalent to another time‐varying exposure model)
Ozen et al.^33^	Cohort/survival	RA/US National databank of rheumatic disease (FORWARD) (18 754)	Cardiovascular events	Glucocorticoids (WCE) + biological DMARDs^c^ (conventional)	Weights from Dixon et al.^2^	Not stated
Rollot et al.^34^	Cohort/survival	Relapsing–remitting multiple sclerosis/France (2285)	Irreversible disability; secondary progression	Disease modifying treatments	20 years +3 year washout	Only WCE used
Josephson et al.^26^	Cohort/survival	Adults with epilepsy/England (50 888)	Cardiovascular disease	Enzyme‐inducing anti‐seizure medications	25 years	Not stated (WCE used for comparison)
Freedman et al.^25^	Cohort/survival	Diabetes/Israel (145 617)	Prostate cancer	Metformin	7 years	Not stated
Kelly et al.^32^	Cohort/survival	Chronic obstructive pulmonary disease/Australia (3523)	Hospitalisation for exacerbation	Paracetamol (acetaminophen)	75 days	Yes
Kedra et al.^22^	Cohort/logistic regression	RA^b^/France (418)	Favourable outcome (disease activity and health assessment)	DMARDs^c^	10 years	Yes

^a^ Propensity score.

^b^ Rheumatoid arthritis.

^c^ Disease modifying anti‐rheumatic drug.

^d^ Nested case–control/conditional logistic regression.

^e^ Inverse probability of treatment weights.

^f^ Human immunodeficiency virus.

^g^ Non‐steroidal anti‐inflammatory drug.

### 3.1 Evolution of the WCE methodology

A brief description of the WCE method can be found in Appendix A.

The first three studies described the WCE method using the association between benzodiazepines and fall related injuries as an example.^8^, ^9^, ^10^ Initially, Abrahamowicz et al.^8^ described a method which used a weight function defined a priori, developed from cumulative exposure methodology in other fields such as environmental exposures^3^, ^5^, ^6^; an exponential decay function (half Gaussian) was chosen using external (clinical) information on the drug half‐life. Subsequently, the authors identified that using a misspecified a priori model can reduce the power to test for an association and proposed that the weight function be estimated using data driven approach with flexible (unpenalised) cubic B‐splines.^9^, ^10^ They validated the WCE models and demonstrated interpretive advantages over other methods of modelling exposure.

The ‘WCE’ R package for time to event outcomes was published in 2015 and is freely available, implemented through a standard Cox proportional hazards model which allows for adjustment of covariates.^11^ Extensions published since 2019 include competing risks survival models^12^, ^13^ and continuous outcomes,^14^ but these are currently not included in the current version of the R package.^11^ Other recent methodological applications of the WCE method include identification of critical time windows^15^ and pharmacovigilance in a data‐driven approach to extract potential adverse events.^16^, ^17^ Additional author‐written extensions to the standard Cox model include inverse probability weighting and marginal structure models.^18^ Three studies^2^, ^19^, ^20^ used a case–control design for computational convenience^21^ (Table 1) and simpler interpretation of the cumulative exposure (see Appendix A). Two studies^22^, ^23^ used a cohort study design with outcomes defined at fixed time intervals and logistic regression for the analysis. A version of the software for regular and conditional logistic regression has not yet been implemented in the current R package. A discrete‐time implementation of the data driven WCE method modelled without splines has been used in some studies with exposure time windows of several years,^24^, ^25^, ^26^ where weights were calculated for time periods of months to years rather than days.

9 of 17 (53%) studies^2^, ^18^, ^19^, ^20^, ^27^, ^28^, ^29^, ^30^, ^31^ using the WCE method with data‐driven weights included the method developers as authors. Of the remaining 8 studies which did not involve the developers,^22^, ^23^, ^24^, ^25^, ^26^, ^32^, ^33^, ^34^ all were published after the release of the R package in 2015.

### 3.2 Comparisons of WCE and conventional models

Model fit is assessed using the Akaike information criterion (AIC) or Bayesian information criterion (BIC), where a difference of 10 points or more is significant and models with a difference of less than 4 points are considered equivalent.^10^ Two studies used the WCE method with weights defined a priori to investigate cerebrovascular disease^35^ or acute myocardial infarction^36^ associated with glucocorticoids in patients with rheumatoid arthritis (Table 1). In both of these studies, the a priori WCE models had worse fit than models combining conventional definitions of exposure with propensity scores. In contrast, 10 studies^2^, ^18^, ^20^, ^22^, ^23^, ^27^, ^29^, ^30^, ^31^, ^32^ that used the flexible WCE spline models found that the WCE method had better fit than conventional models and one found the WCE model fit was considered equivalent to conventional models.^19^ The remaining six studies^24^, ^25^, ^26^, ^28^, ^33^, ^34^ did not report on model fit comparisons with conventional models. One possible reason for the lack of comparison is that as the WCE method becomes more mature and widely used, there is less of a perceived need to justify its use compared to conventional models in articles published more recently. In addition, the WCE model is able to reproduce the effect of exposure from several simpler conventional models.^10^

### 3.3 Clinical applications

Following the initial methodological articles investigating benzodiazepines and falls, as described above, there were 19 clinical applications identified which met our selection criteria (Table 1). Both adverse and beneficial outcomes were investigated. Two early studies used the a priori WCE method, while 17 used data driven methods to determine the weights, including flexible cubic splines. There was an early focus on adverse outcomes from glucocorticoids in patients with rheumatoid arthritis.^2^, ^23^, ^29^, ^35^, ^36^ Since 2018, the method has been applied to a wide range of cohorts, exposures and outcomes, particularly for the long‐term effect of exposure: 13 studies used time windows of 1 year or longer. Several studies have investigated adverse or beneficial outcomes from disease modifying antirheumatic drugs (DMARDs) in autoimmune or rheumatic conditions such as rheumatoid arthritis^22^, ^23^, ^28^, ^33^ or multiple sclerosis.^34^ The benefit from bevacizumab in overall survival of patients with metastatic colorectal cancer in a randomised controlled trial was re‐analysed using the WCE method.^27^

There were three studies investigating adverse events, such as cancer, from metformin in diabetes patients.^24^, ^25^, ^31^ Other studies investigated myocardial infarction and NSAIDs,^19^ anti‐seizure medicines and cardiovascular disease,^26^ exacerbation of chronic obstructive pulmonary disease and paracetamol^32^ or the timing of antibiotic resistance after exposure to ciprofloxin.^20^

### 3.4 Estimation of the time window

Choice of an appropriate time window for assessing exposure may be informed by several factors such as drug pharmacology or pharmacokinetics, typical prescribing patterns and time frames of outcomes following exposure. For example, Abrahamowicz et al.^10^ used the 40–100 h half‐life of flurazepam to select a 30‐day window for the risk of falls. The weight function showed that doses taken prior to 10 days had no impact on the outcome, as the authors expected. In contrast, due to uncertainty in the time frames for the association between infection and long‐term glucocorticoid use, Dixon et al.^2^ compared WCE models with time windows of 1–3 months, 1–4 years, fitted with splines of between 1 and 3 interior knots. This choice was appropriate since glucocorticoids can be taken continuously or intermittently over a period of several years and infection occurred after a mean follow‐up period of 3.8 years in their study. The WCE model with a 3‐year time window and 1 knot was the best fit to the data. The authors found that although the most recent doses had the highest weights and therefore the most impact on the risk of infection, doses up to 2.5 years previously were also associated with current infection risk.

### 3.5 Strengths and limitations of the WCE spline method

The main strength of the WCE method is the aetiologically and biologically relevant modelling of exposure that accounts for intensity, timing and duration (Table 2). This is particularly relevant for time‐varying exposures of long duration, such as glucocorticoids or DMARDs, which were the subject of 9 studies. The weight function may be used to determine potential causal relationships between the exposure and outcome, for example adaptive immunity from doses in the past 1 to 3 years compared with impaired innate immunity from recent doses in glucocorticoid therapy.^2^

**TABLE 2:** Strengths and limitations of the WCE spline‐based method and its implementation in the R package ‘WCE’ version 1.0.2.

Strengths	Limitations
Accounts for intensity, timing and duration of exposure	R package calculates weight function for one exposure effect without possible interactions or accounting for drug switching
Positive and negative weights model adverse/protective effect of drug at different time periods	R package for simple Cox models only. Needs to be modified to account for more complicated models (e.g. stratified Cox models, IPTW)
Aetiologically relevant effect of cumulative exposure on outcome	May be subject to overfit bias, especially for exposures further in the past
Combines effect of pharmacokinetics, pharmacodynamics and biological mechanisms	Interpretation of the weight function requires more care, especially for mix of positive and negative weights
No prior assumptions about shape of the weight function and the flexibility of the weight function can be changed by varying the number of interior knots	Spline fit to sharp changes in the weight function require manual placing of knots in areas of large curvature
Ability to predict outcome for varying patterns of exposure	Instability of spline function near time zero where the association between exposure and outcome is null or weak
Different time windows for aetiologically relevant exposures allowed	Selection of appropriate of best time window may be unclear according to current guidelines

Another strength is the data driven nature of the spline function fit; no a priori weight function is assumed, with weights quantifying the relative importance of exposure at different times in the past. In addition, weights are not constrained to be either positive or negative, allowing them to reflect either a positive or negative association between exposure and outcome. The weight function can be unconstrained or constrained to be zero the start or end of the time window; all the included studies assumed the weight function smoothly decayed to zero at the end of the time window, which is biologically plausible in pharmacoepidemiology.

While allowing for different biologically plausible exposure time windows is a strength of the method, selecting the most appropriate time window may not be straightforward. The guidelines^9^, ^10^ state that the model with the lowest AIC or BIC should be chosen but that models with a difference of less than 4 points are considered equivalent. Thus, if the difference in AIC or BIC between models is small, subjective judgement is required and it may be difficult to select the best model, either between different WCE time windows^20^ or between WCE and other models.^19^, ^27^ When comparing several drugs from the same class, using the same time window for the weight functions may be preferred for comparison purposes,^19^ even if it may not be the best fitting for each drug. Selecting an appropriate time window may require additional clinical information such as pharmacodynamics, pharmacokinetics or latency periods between drug initiation and outcome.^9^ In addition, the weight function may be difficult to interpret clinically, for example, time intervals containing negative weights with confidence bands below zero have been interpreted either as a protective effect^23^, ^32^ or recent a withdrawal effect when negative weights switch to positive for exposures further in the past.^10^

When the association between exposure and outcome is weak or null, the spline fit may be unstable, especially near time zero, making it difficult to determine if there is a true association and if there is, whether it is positive or negative.^10^ Additionally, the spline fit in time intervals where there is little data, usually for exposures further in the past, may lead to overfit bias; however the 95% confidence bands can inform the true association in these time intervals.^10^

Some limitations arise from the basic Cox proportional hazards model implemented in the R package. Testing the proportional hazards assumption, or more complicated applications require modification to the code, such as incorporating marginal structure models,^18^ stratified Cox proportional hazards models^32^ or using competing risks.^12^, ^13^

## 4 DISCUSSION

Although the concept of WCE has existed for some decades, development of freely available statistical software has facilitated recent applications of the method. The increase in the number of papers after the software was released demonstrates that alternative exposure representations are gaining familiarity in pharmacoepidemiology.

The WCE method is well suited to exposure‐outcome studies where exposures at different times in the past may have different effects on the risk, particularly in long‐term exposures. Use of the WCE method allows the potential discovery of important associations that are not possible using conventional exposure definitions, such as in a study of enzyme inducing anti‐seizure drugs in epilepsy and long‐term cardiovascular disease.^26^ The shape of the weight function and the best fitting time window provides additional insights into the nature of the association, the biological latency period and how the effects of exposure accumulate over time^19^ and we have recommended that the plot of the weight function is always published. For example, in studies of flurazepam and falls^10^ and paracetamol and COPD exacerbation,^32^ the weight function varied between negative and positive. Negative weights shortly after initiation produced exposure‐outcome associations which were protective but less than a week later, positive weights represented harmful effects. Negative followed by positive weights also translated into a short‐term withdrawal effect soon after stopping which disappeared after a few days. Knowledge of temporal risk is important in clinical decision making, for example when to stop glucocorticoids before elective surgery to reduce the risk of infection, or what dose and duration of high intensity glucocorticoid therapy does not elevate infection risk.^2^

In practice, there may be technical difficulties in implementing the WCE method, including calculating the daily dose from administrative data,^1^ the choice of the time window, testing the proportional hazards assumption or adapting the software to more complicated regression models. Implementation of the method may add an extra layer of complexity to studies; most applications presented additional information on the choice of weight function and comparisons with conventional models, often in supplementary material. Comparisons of the WCE models with conventional models using alternative definitions of exposure or sensitivity analyses may provide additional insight into whether a true association exists.^31^ Combining alternative models of exposure has been recommended to improve the timeliness of signal detection of adverse events.^37^

Cadarette et al.^38^ described five attributes that affect the adoption of an innovation: (1) relative advantage; (2) compatibility with needs of potential users; (3) complexity; (4) trialability or testability; and (5) observability of results to others. While the WCE method demonstrates the first and fourth attributes, improving the remaining attributes may improve uptake of the method. The method developers were authors in more than half the studies using the WCE approach, suggesting they played an important role in diffusion of the new method.^38^ Although use of the method increased after the release of the R package, including studies not involving the developers, the rate of increase is still limited. This may be due to unfamiliarity with the method, its additional complexity and restriction of the current WCE package to time to event outcomes, which are not compatible with methods required by some studies.^38^ A version of the package with additional features may overcome some of these challenges.

### 4.1 Suggested recommendations

Following the literature review, we make the following recommendations for using the WCE method and suggestions for enhancements of the software:

- Follow author guidelines^9^, ^10^ for using and reporting the method in the published results:
  - A comparison of WCE models with different clinically relevant time windows.
  - A comparison of WCE model fit with conventional models.
  - A plot of the weight function for the selected final WCE model.
- Recommendations to select the best time window in the case of models with an equivalent fit to the data:
  - If the AIC and BIC differ in the choice of best fitting model, use the AIC since simulations showed it was better at identifying the correct model, particularly if there were >500 events.^10^
  - Use clinical judgement to select the weight function with the most plausible shape.
- Software enhancements could include:
  - Publicly available versions of the software that allow for stratified, weighted Cox models, exposure‐confounder interactions and robust standard errors to account for clustering since these are commonly used in pharmacoepidemiology.
  - Conditional logistic regression models for nested case–control studies and logistic regression for cohort studies.

We noted that articles which included authorship by one or more of the method developers compared WCE and conventional models and published the plot of the weight function, but articles published after the release of the ‘WCE’ package did not always follow these guidelines. Consistent execution and reporting of the WCE method, including items in Table 3, would facilitate interpretation of the results and allow for meaningful comparison of similar studies.

**TABLE 3:** Checklist for reporting of the WCE method to be used in conjunction with STROBE guidelines.

Item	Completed
Definition of exposure	☐
Description of time windows assessed	☐
Comparison of model fit with conventional models	☐
Plot of weight function	☐

## 5 CONCLUSION

The WCE method is an important tool for exploring the effect of time‐varying exposures on an outcome, including the dose, duration, and timing of past exposures. The flexible nature of the spline fit allows the association to change over time elapsed since exposure and investigate the effect of short‐ or long‐term exposure, which provides insights into biological latency periods and the prediction of the effect of different exposure patterns on the outcome. The additional complexity of its implementation, however, makes it more difficult to use for hypothesis‐free data mining of exposure‐outcome pairs. Despite this, the intuitive concept of WCE is a powerful addition to conventional methods of classifying exposure; however consistent execution and reporting will facilitate transparent interpretation and enable a robust comparison of findings from similar studies, which will increase familiarity with the method and aid diffusion to other groups of researchers. Software enhancements which incorporate additional analysis methods for different study designs may increase the rate of uptake of the method.

The WCE method is an appropriate choice when the research question involves assessment of risk from cumulative exposure, or when timing of the exposure may affect current risk. It can be used with common study designs and is an important addition to the pharmacoepidemiology toolbox alongside conventional definitions of exposure.

## FUNDING INFORMATION

This study was supported by the Australian Government National Health and Medical Research Council, grant numbers APP1040938 and GNT1157506.

## CONFLICT OF INTEREST STATEMENT

The authors declare no conflict of interest.

## ETHICS STATEMENT

The authors declare that no ethics approval was needed for this study

## Appendix A DESCRIPTION OF THE WCE METHOD

### A.1

The weight function $w\left(u-t\right)$ assigns weights to exposures in the past at time t, where current time during follow‐up is denoted as uand t≤u. The weighted cumulative exposure $\mathrm{WCE}\left(u\right)$ at timeu from past exposures $X\left(t\right)$ is defined as

$$\mathrm{WCE}\left(u\right)=\sum_{t}^{u}w\left(u-t\right)X\left(t\right)$$

Modelling the effect of past exposures through the WCE method is a convolution, with the weight function as the kernel.^9^ ‘Time zero’ in the weight function occurs when t=u; that is, on the current day of follow‐up.

Weighted cumulative exposure $\mathrm{WCE}\left(u\right)$ is used in place of a conventional exposure covariate in regression modelling. If the proportional hazards assumption for weighted cumulative exposure is valid, the Cox proportional hazards regression model with no additional covariates is:

$$h\left(u|X\left(u\right)\right)=h_{0}\left(u\right)\exp\left[\beta \mathrm{WCE}\left(u\right)\right]=h_{0}\left(u\right)\exp\left[\beta \sum_{t}^{u}w\left(u-t\right)X\left(t\right)\right]$$

where $h\left(u|X\left(u\right)\right)$ is the instantaneous risk at follow‐up time u from past exposures $X\left(u\right)$ and $h_{0}$ is the baseline hazard (not estimated).

Hauptmann et al.^4^ and Sylvestre and Abrahamowicz^9^ proposed estimating the weight function as the sum of m (unpenalised) cubic regression B‐splines $B_{j}\left(t\right)$:

$$w\left(u-t\right)=\sum_{j=1}^{m}\theta_{j}B_{j}\left(u-t\right)$$

In the Cox proportion hazards model, the WCE is included as artificial time varying covariates $D_{j}\left(u\right)=\sum_{t}^{u}B_{j}\left(u-t\right)X\left(t\right)$:

$$h\left(u|X\left(u\right)\right)=h_{0}\left(u\right)\exp\left[\sum_{j=1}^{m}\gamma_{j}D_{j}\left(u\right)\right]$$

where $\gamma_{j}=\beta \theta_{j},j=1,\ldots ,m$

The usual methods can be used to test the proportional hazards assumption for the artificial time varying covariates $D_{j}\left(u\right)$, for example using weighted Schoenfeld residuals.

The exposure at any time u during follow‐up can be modelled as the weighted cumulative exposure of the previous a days, where a is an aetiologically relevant time window. The following process has been implemented in the R package ‘WCE’^11^ to fit flexible cubic splines to the weighted cumulative exposure in Cox regression models:

1. For each event, calculate for every person in the risk set their artificial time varying covariates $D_{j}\left(u\right)$ for the cumulative daily dose to the end of the selected time window a
2. Add $D_{j}\left(u\right)$ in the Cox model
3. Use the estimated coefficients from the Cox model to calculate the weight function.
4. The estimated weight function can be used to predict the hazard ratio from a given exposure pattern compared with a reference.

The analyst repeats the process for different time windows a and uses the AIC or BIC to select the time window that produces the best fitting model. A difference of more than 10 points is considered significant while models within 4 points are considered equivalent.^10^

Weight functions can be constrained or unconstrained at either end of the chosen time window a. Constrained models assume that the effect of exposure on the outcome smoothly reach zero at the end of the time window. Constrained and unconstrained models assume the effect is null after periods that are longer than the time window. All applied studies reviewed for this article used constrained models. The flexibility of the weight function can be controlled by varying the number of knots, with one interior knot commonly used.

For nested case–control studies, conditional logistic regression replaces the Cox proportional hazards model.^4^ The WCE is modelled in different ways for time to event (survival) analysis and case control studies. In time to event analysis, due to the proportional hazards assumption, exposure at any time u during follow‐up is the weighted cumulative exposure of the previous a days. For case–control studies, exposure is modelled as the weighted cumulative exposure of the previous a days before the index date only.^3^, ^9^
